# Stereotactic ablative radiotherapy versus conventionally fractionated radiotherapy in the treatment of hepatocellular carcinoma with portal vein invasion: a retrospective analysis

**DOI:** 10.1186/s13014-019-1382-1

**Published:** 2019-10-22

**Authors:** Jen-Fu Yang, Cheng-Hsiang Lo, Meei-Shyuan Lee, Chun-Shu Lin, Yang-Hong Dai, Po-Chien Shen, Hsing-Lung Chao, Wen-Yen Huang

**Affiliations:** 1 Department of Radiation Oncology, Tri-Service General Hospital, National Defense Medical Center, No. 325, Sec. 2, Cheng-Kong Rd. Nei-Hu, 11490 Taipei, Taiwan; 2 School of Public Health, National Defense Medical Center, Taipei, Taiwan; 3 Institute of Clinical Medicine, National Yang-Ming University, Taipei, Taiwan

**Keywords:** Hepatocellular carcinoma, Portal vein invasion, Portal vein thrombosis, Stereotactic ablative radiotherapy, Stereotactic body radiotherapy, Conventionally fractionated radiotherapy

## Abstract

**Background:**

This study aimed to compare the clinical outcomes of stereotactic ablative radiotherapy (SABR) and conventionally fractionated radiotherapy (CFRT) in hepatocellular carcinoma (HCC) patients with portal vein invasion (PVI).

**Methods:**

HCC patients with PVI treated with radiotherapy from 2007 to 2016 were analysed. CFRT was administered at a median dose of 51.5 Gy (interquartile range, 45–54 Gy) with 1.8–3 Gy per fraction. SABR was administered at a median dose of 45 Gy (interquartile range, 40–48 Gy) with 6–12.5 Gy per fraction. Treatment efficacy, toxicity, and associated predictors were assessed.

**Results:**

Among the 104 evaluable patients (45 in the SABR group and 59 in the CFRT group), the overall response rate (ORR, complete and partial response) was significantly higher in the SABR group than the CFRT group (62.2% vs. 33.8%, *p* = 0.003). The 1-year overall survival (OS) rate (34.9% vs. 15.3%, *p* = 0.012) and in-field progression-free survival (IFPS) rate (69.6% vs. 32.2%, *p* = 0.007) were also significantly higher in the SABR vs. CFRT group. All 3 rates remained higher in the SABR group after propensity score matching. Multivariable analysis identified SABR and a biologically effective dose ≥65 Gy as favourable predicators of OS. There was no difference between treatment groups in the incidence of radiation-induced liver disease or increase of Child-Pugh score ≥ 2 within 3 months of radiotherapy.

**Conclusions:**

SABR was superior to CFRT in terms of ORR, OS, and IFPS. We suggest that SABR should be the preferred technique for HCC patients with PVI.

## Background

Portal vein invasion (PVI) frequently develops in patients with advanced hepatocellular carcinoma (HCC), and has an estimated incidence rate of 34 to 80% [1, 2]. Without treatment, PVI prognosticates extremely poor prognosis with a median survival of only 2.7 months [3]. Sorafenib is currently regarded as the standard systemic therapy for HCC with PVI, but the survival gain is only 2–3 months [4–7].

For various locoregional modalities including surgical resection, transarterial chemoembolization (TACE), transarterial radioembolization, hepatic artery infusion chemotherapy attempted in patients with PVI, only carefully selected patients are amendable. Radiotherapy (RT) presents the only noninvasive alternative which is not dependent on vasculature to access the tumor, and is therefore not associated with a risk of hepatic ischemia. Over the past few decades, the development of the three-dimensional conformal technique has allowed for partial liver irradiation. Studies of conventionally fractionated radiotherapy (CFRT) in PVI have shown improved outcomes, with 1-year overall survival (OS) of 16.7–40.2% and overall response rates (ORR) of 23.5–45% [8–12].

Stereotactic ablative radiotherapy (SABR) is an emerging technique, and can achieve tumorcidal doses in limited fractions, with significant normal tissue sparing. Several studies have reported favorable results with SABR for treating different cancers [13, 14]. We hypothesized that SABR would provide more benefit than would CFRT in HCC patients with PVI.

The aim of this study was to present a single-institutional experience with a relatively large number of patients, and to compare the difference in clinical outcomes between the two RT techniques.

## Methods

### Patients

After obtaining waiver of consent for this retrospective research from the institutional review board of Tri-Service General Hospital (approval number: 1–107–05-016), we identified HCC patients with PVI undergoing RT from January 2007 to December 2016. Patients with Vp3/Vp4 invasion (invading the first-order branches and/or main trunk of the portal vein), an Eastern Cooperative Oncology Group (ECOG) performance status of 0 to 2 and a Child-Pugh (CP) class of A or B were eligible. Any prior interventions were allowed, except for previous RT. Those patients were considered unsuitable for standard locoregional treatment via multidisciplinary committee discussion. Given increasing evidence of the benefits of SABR for HCC patients in the past decades, our hospital practice naturally shifted from use of CFRT to use of SABR. Because SABR was not reimbursed until 2015 in Taiwan, the choice of RT technique was determined partly by the patients’ financial resources.

Diagnosis of HCC was made either by biopsy or by radiologic investigation based on characteristic imaging findings [15]. PVI was confirmed as a low-attenuation intraluminal mass that expanded the portal vein on contrast-enhanced helical computed tomography (CT) scans or on magnetic resonance imaging (MRI) scans. Pretreatment evaluation consisted of medical history, physical examination, complete blood counts, serum biochemistries, alpha-fetoprotein (AFP) level, chest film, and MRI and/or CT of the abdomen. Bone scan, positron emission tomography, or liver angiography was performed if clinically indicated.

### CFRT technique

**Fig. 1:**
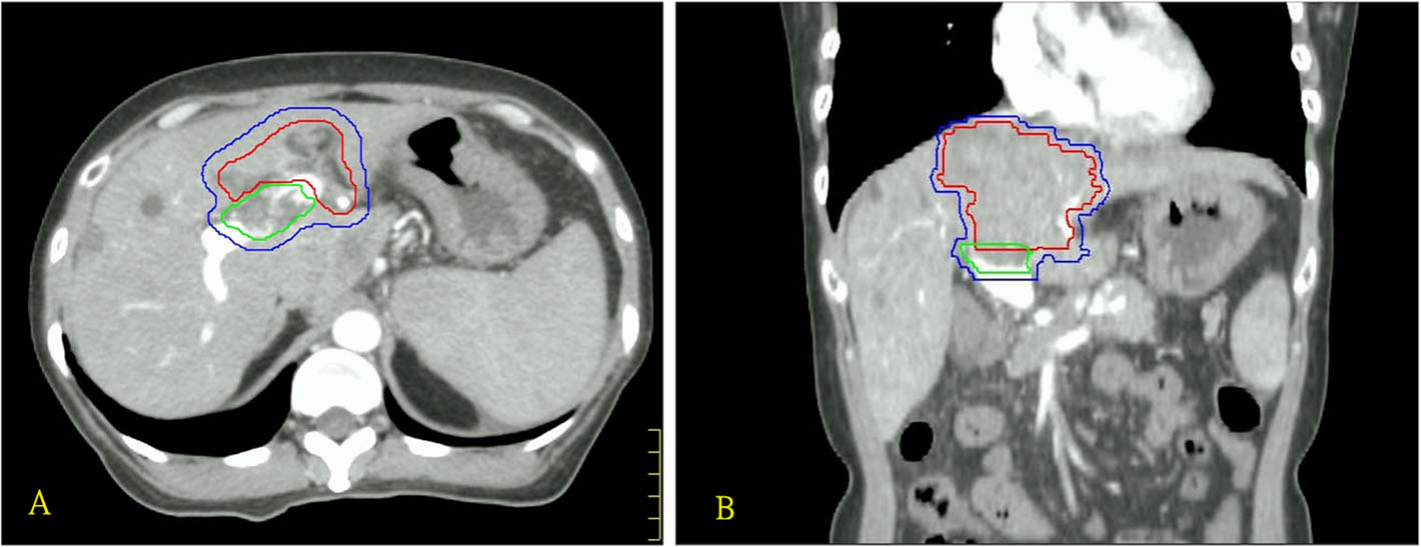
Example of target delineation in the axial (**a**) and coronal (**b**) view of computed tomography scans. The green, red, and blue lines represent detectable portal vein invasion, adjacent tumor, and clinical target volume, respectively

For better delineation of the upper gastrointestinal tract, most patients were asked to take oral contrast medium before simulation. A non-contrast CT simulation with a 3 mm slice thickness was performed. During the scanning procedure, all patients were supine, and were immobilized with a vacuum cushion, with arms raised overhead. Motion management with four-dimensional CT was conducted in some patients. Breath-holding was not mandatory. Either three dimensional conformal radiotherapy (3DCRT) or intensity-modulated radiotherapy was designated, depending on the physicians’ discretion, using the Nucletron Plato RTS v2.6.3 planning system, which was replaced by Philips Pinnacle v9.0 planning system in 2009. Instead of defining a gross target volume, the clinical target volume (CTV) was contoured directly. Contrast images registration was used to assist CTV delineation. The CTV was defined as a margin of 3–5 mm around the detectable PVI. Partial hepatic tumor was included in the CTV if the primary hepatic tumor was near to the detectable PVI or if the portal vein was invaded by the tumor directly. An example of target delineation is shown in Fig. 1. The planning target volume (PTV) was generated by expanding the CTV by 8 mm radially and by 10–15 mm craniocaudaully. Prescribed dose to the PTV was 45–54 Gy (1.8–3 Gy per fraction, five fractions per week), administered with a 15 MV photon beam. Most PTV was encompassed in the 90% isodose curve. The irradiation field contained 3–6 gantry angles. RT was delivered using an Elekta Precise or a Siemens Primus linear accelerator.

### SABR technique

SABR was administered using the CyberKnife radiosurgery system (Accuray, Sunnyvale, CA), delivering 6 MV photons. At least one week before CT simulation, 4–6 fiducial markers were placed within or around the tumor under sonographic or CT guidance. An individually shaped vacuum pillow allowed patient immobilization in the supine position, with a vest for synchrony tracking and with abdominal compression devices to reduce respiratory motion. A contrast CT scan with a 1 mm slice thickness was obtained for treatment planning with or without MRI scan registration. The definition of CTV in SABR was the same as in the CFRT technique. The PTV was defined as the volume with a margin of 0–3 mm added to CTV for patients with fiducial implantation. If fiducial implantation failed or was unsuitable, the PTV margin was expanded to 8–20 mm craniocaudally, based on the liver motion. Margin modification was permitted for respecting normal tissue tolerance. Prior to August 2009, the MultiPlan CyberKnife treatment planning system version 1.7.0 was employed for treatment planning, and was updated to version 2.1.0 thereafter. The median total dose was 45 Gy, at 6–12.5 Gy per fraction, with 4 to 5 fractions administered on consecutive working days. The detailed dose-limiting organs and their constraints are described in our previous publication [16].

### Evaluation and follow up

All patients were seen at least once per week during RT, 1–2 months for the first six months after RT, and every three months thereafter. Image evaluation with CT or MRI was obtained every 1–3 months. Modified Response Evaluation Criteria in Solid Tumors was used for evaluation of the PVI response [17].

Hepatic toxicity assessment consisted of Radiation-induced liver disease (RILD) and increase of CP score ≥ 2. Only patients with either adequate follow-up of three months or death and/or occurrence of toxicity within 3 months were included in the analysis. Physical examinations and blood tests conducted at every visit, were used for toxicity assessment. RILD was defined as either classic or non-classic, without intrahepatic tumor progression noted within three months after RT. Classic RILD manifested as the presence of nonmalignant ascites and the elevation of anicteric alkaline phosphatase level to at least twice the upper normal values. Non-classic RILD manifested as the elevation of transaminase levels to at least five times the upper limit of the normal or pre-treatment values.

### Statistics

Doses were converted to biologically effective doses (BED) with an α/β ratio of 10 for analysis. Between-group comparisons were conducted by chi-square tests or by Student’s t-tests as appropriate. OS was measured from the first day of RT until the date of death from any cause or last follow-up. In-field progression-free survival (IFPS) was measured from the first day of RT until the date of tumor recurrence or progression in irradiated field or last follow-up and patients who have received local intervention (ex, surgery or reirradiation) were censored at the date of procedure. Kaplan Meier curves were constructed for OS and IFPS, and the difference was compared using the log-rank test. The Cox Proportional Hazards model was applied to identify potential predictors of survival. A receiver operating characteristic (ROC) curve was used to define optimal cut-off points for continuous variables based on the Youden index. Given the imbalance of potential confounders between groups, propensity score-matching was used. Propensity scores were estimated from a logistic regression model that included treatment period, sex, age, prior treatment, virus infection type, performance, tumor size, tumor number, extrahepatic metastasis, PVI location, AFP level, CP class, and sorafenib use. A nearest neighbor method with a caliper width of 0.2 was used to create matched cohorts. A 2-tailed *p* < 0.05 was designated as statistically significant for all tests except when variables with *p* < 0.10 in the univariable Cox model were entered into a multivariable Cox model. SPSS (SPSS Inc. Chicago, IL, USA) version 22 and R statistical software version 3.4.3 (R Foundation, https://www.r-project.org)) were used for data analysis.

## Results

### Patient characteristics

**Fig. 2:**
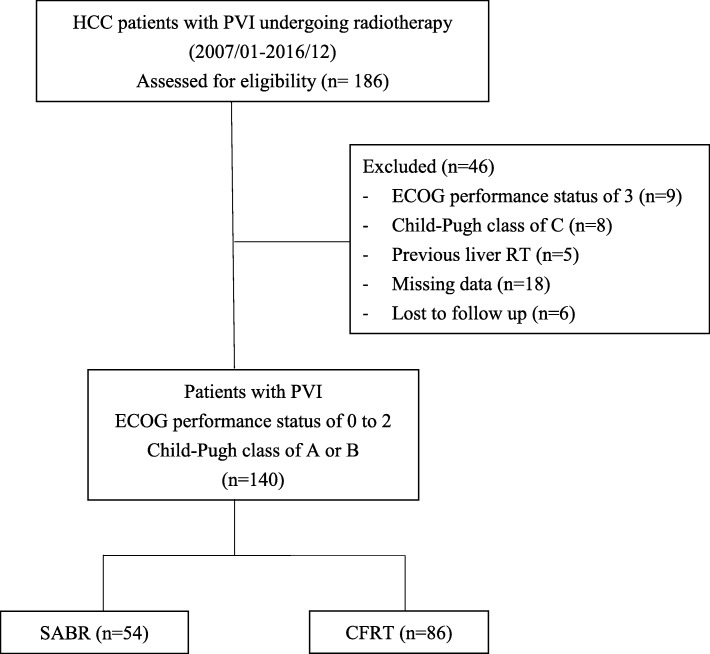
Flow diagram for the selection of hepatocellular carcinoma patients with portal vein invasion

Initially, 186 patients were identified by chart review. Forty-six patients were excluded owing to ineligibility, missing data or loss of follow-up after RT (Fig. 2). Finally, 140 patients were entered for data analysis. SABR was administered to 54 patients (median dose, 45 Gy; inter-quartile range [IQR], 40–48 Gy; 6–12.5 Gy per fraction), and CFRT was administered to 86 patients (median dose, 51.5; IQR, 45–54 Gy; 1.8–3 Gy per fraction). Detailed patient characteristics are listed in Table 1.

**Table 1:** Patient characteristics of the entire cohort

	SABR (*n* = 54)	CFRT (*n* = 86)		
Variable	No. of patients (%)	No. of patients (%)	*p*-value	SMD
Time of treatment			0.789	0.047
Before December 31, 2011	27 (50.0)	45 (52.3)		
After December 31, 2011	27 (50.0)	41 (47.7)		
Age, year			0.494	0.243
Mean (SD)*, range	61.0 (12.9), 32–84	59.6 (11.2), 34–90		
≤ 60	23 (42.6)	47 (54.7)		
> 60	31 (57.4)	39 (45.3)		
Sex			0.766	0.052
Male	42 (77.8)	65 (75.6)		
Female	12 (22.2)	21 (24.4)		
Liver disease			0.281	0.086
HBV	29 (53.7)	56 (65.1)		
HCV	17 (31.5)	16 (18.6)		
HBV and HCV	1 (1.9)	4 (4.7)		
Non-virus	7 (13.0)	10 (11.6)		
ECOG			0.887	0.025
0–1	46 (85.2)	74 (86.0)		
2	8 (14.8)	12 (14.0)		
Extrahepatic metastasis			0.993	0.001
Yes	5 (9.3)	8 (9.3)		
No	49 (90.7)	78 (90.7)		
AFP, ng/ml			0.960	0.009
≤200	23 (42.6)	37 (43.0)		
> 200	31 (57.4)	49 (57.0)		
Child-Pugh class			0.086	0.303
A	35 (64.8)	43 (50.0)		
B	19 (35.2)	43 (50.0)		
Prior treatment			0.047	0.350
Yes	30 (55.6)	33 (38.4)		
No	24 (44.4)	53 (61.6)		
No. of tumor			0.894	0.023
Multiple	37 (68.5)	58 (67.4)		
Single	17 (31.5)	28 (32.6)		
Tumor size, cm			0.088	0.298
≤8	28 (51.9)	32 (37.2)		
> 8	26 (48.1)	54 (62.8)		
PVI location			0.128	0.267
Vp4	23 (42.6)	48 (55.8)		
Vp3	31 (57.4)	38 (44.2)		
Sorafenib			0.825	0.038
Yes	23 (42.6)	35 (40.7)		
No	31 (57.4)	51 (59.3)		
BED, Gy			< 0.001	1.075
< 65	14 (25.9)	63 (73.3)		
≥ 65	40 (74.1)	23 (26.7)		

*Abbreviations: SABR* Stereotactic ablative radiotherapy, *CFRT* Conventionally fractionated radiotherapy, *HBV* Hepatitis B virus, *HCV* Hepatitis C virus, *ECOG* Eastern Cooperative Oncology Group, *AFP* Alpha fetoprotein, *PVI* Portal vein invasion, *BED* Biologically effective dose, *SD* Standard deviation, *SMD* Standardized mean difference. *t-test

### PVI response

Follow-up images were available for 45 SABR patients and for 59 CFRT patients (83.3% vs. 68.6%, *p* = 0.052). Among evaluable patients, five complete response, 23 partial response, 15 stable disease, and two progressive disease cases were observed in the SABR group, and five complete response, 15 partial response, 27 stable disease, and 12 progressive disease cases were observed in the CFRT group. The ORR (complete and partial response) was significantly higher in the SABR group than it was in the CFRT group (62.2% vs. 33.9%, *p* = 0.004). Of all patients, 18 in the SABR group and 13 in the CFRT group were able to achieve either complete or partial recanalization of the invaded vein (33.3% vs. 15.1%, *p* = 0.012); subsequent TACE was conducted in 9 patients of the SABR group and in 11 patients of the CFRT group (16.7% vs. 12.8%, *p* = 0.524).

### Survival

**Fig. 3:**
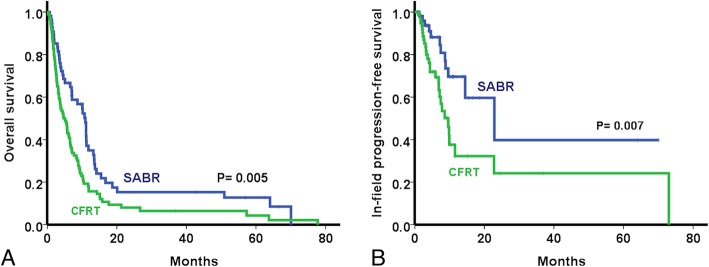
Overall survival (**a**) and in-field progression-free survival (**b**) in the entire cohort using Kaplan Meier method

The median follow-up period was 6.2 months for all patients and 15.4 months for those alive. At the time of the analysis, nine patients in the SABR group and four patients in the CFRT group were alive. Before propensity score matching, the median survival was 10.9 months in the SABR group and 4.7 months in the CFRT group. The 1- and 2-year OS rates were 34.9% and 15.3% in the SABR group, and 15.7% and 8.0% in the CFRT group, respectively (*p* = 0.005, Fig. 3a). The 1- and 2-year IFPSs were also significantly higher in the SABR group compared to those in the CFRT group (69.6% vs. 39.8 and 32.2% vs. 24.2%, respectively; *p* = 0.007; Fig. 3b).

**Fig. 4:**
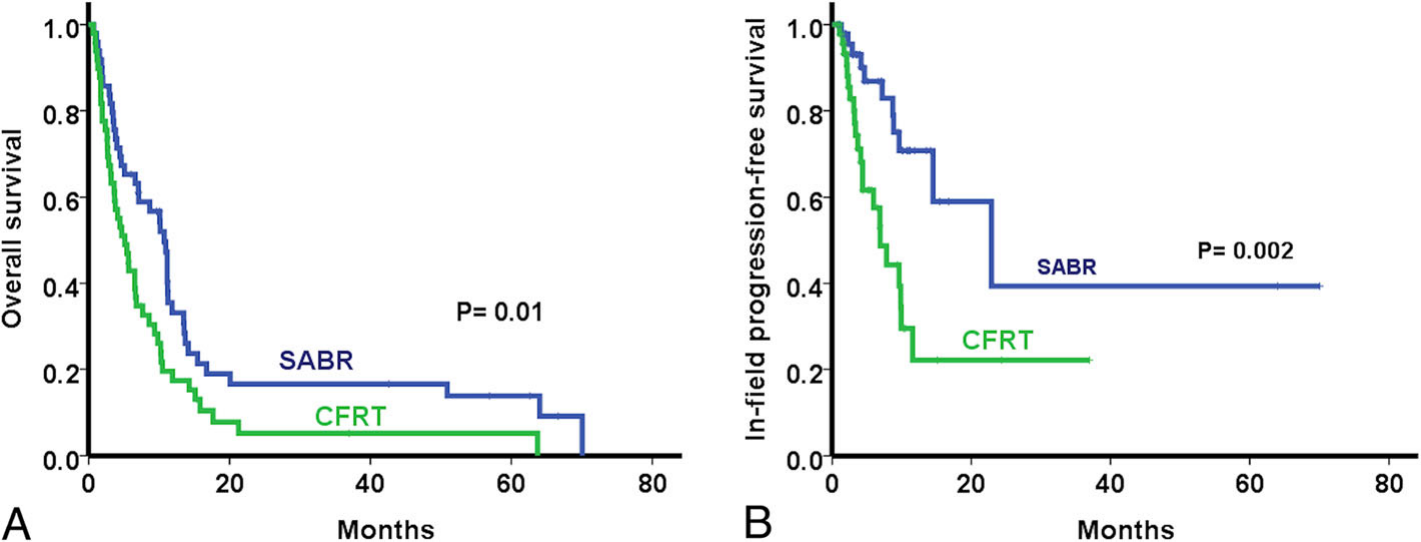
Overall survival (**a**) and in-field progression-free survival (**b**) in the propensity score-matched cohort using Kaplan Meier method

After propensity score-matching, 49 patients in each group were matched. (Table 2). The median survival was 10.7 months in the SABR group and 5.1 months in the CFRT group. The 1- and 2-year OS and IFPSs of the SABR group were significantly higher than those of the CFRT group (OS: 33.1% vs. 16.5% and 17.3% vs. 5.2%, *p* = 0.01, respectively; Fig. 4a; IFPS: 70.8% vs. 39.3% and 22.2% vs. 22.2%, *p* = 0.002, respectively; Fig. 4b).

**Table 2:** Patient characteristics of the propensity score-matched cohort

	SABR (*n* = 49)	CFRT (*n* = 49)		
Variable	No. of patients (%)	No. of patients (%)	*p*-value	SMD
Time of treatment			0.840	0.041
Before December 31, 2011	24 (49.0)	23 (46.9)		
After December 31, 2011	25 (51.0)	26 (53.1)		
Age, year			0.648	0.082
Mean (SD)*, range	60.2 (13.2), 32–84	59.1 (10.9), 34–78		
≤ 60	22 (44.9)	24 (49.0)		
> 60	27 (55.1)	25 (51.0)		
Sex			0.812	0.048
Male	38 (77.6)	37 (75.5)		
Female	11 (22.4)	12 (24.5)		
Liver disease			0.259	0.116
HBV	28 (57.1)	32 (65.3)		
HCV	14 (28.6)	7 (14.3)		
HBV and HCV	1 (2.0)	3 (6.1)		
Non-virus	6 (12.2)	7 (14.3)		
ECOG			0.790	0.054
0–1	41 (83.7)	40 (81.6)		
2	8 (16.3)	9 (18.4)		
Extrahepatic metastasis			1.000	< 0.001
Yes	5 (10.2)	5 (10.2)		
No	44 (89.8)	44 (89.8)		
AFP, ng/ml			0.541	0.124
≤200	20 (40.8)	23 (46.9)		
> 200	29 (59.2)	26 (53.1)		
Child-Pugh class			0.835	0.042
A	31 (63.3)	30 (61.2)		
B	18 (36.7)	19 (38.8)		
Prior treatment				
Yes	25 (51.0)	28 (57.1)	0.543	0.123
No	24 (49.0)	21 (42.9)		
No. of tumor			0.828	0.044
Multiple	34 (69.4)	33 (67.3)		
Single	15 (30.6)	16 (32.7)		
Tumor size, cm			1.000	< 0.001
≤8	24 (49.0)	24 (49.0)		
> 8	25 (51.0)	25 (51.0)		
PVI location			1.000	< 0.001
Vp4	21 (42.9)	21 (42.9)		
Vp3	28 (57.1)	28 (57.1)		
Sorafenib			1.000	< 0.001
Yes	21 (42.9)	21 (42.9)		
No	28 (57.1)	28 (57.1)		
BED, Gy			< 0.001	1.124
< 65	13 (26.5)	37 (75.5)		
≥ 65	36 (73.5)	12 (24.5)		

*Abbreviations: SABR* Stereotactic ablative radiotherapy, *CFRT* Conventionally fractionated radiotherapy, *HBV* Hepatitis B virus, *HCV* Hepatitis C virus, *ECOG* Eastern Cooperative Oncology Group, *AFP* Alpha fetoprotein, *PVI* Portal vein invasion, *BED* Biologically effective dose, *SD* Standard deviation, *SMD* Standardized mean difference. *t-test

### Predictors for survival

**Fig. 5:**
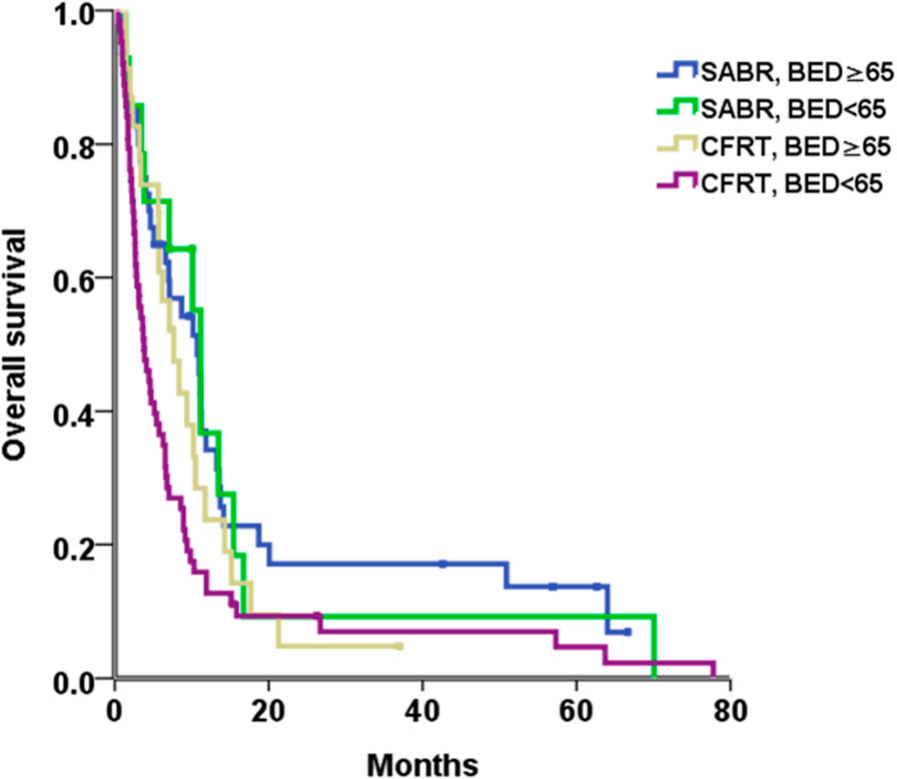
Overall survival considering the biologically effective dose in the entire cohort

Univariable analysis revealed that the presence of SABR, ECOG 0–1, CP class A, single tumor, tumor size ≤8 cm, Vp3, AFP ≤200 ng/ml, prior treatment, and BED ≥65 Gy were predictors of superior OS. No survival difference was noted between patients treated before December 31, 2011 versus after December 31, 2011. Given the presence of the high correlation between BED and the RT technique (*p* < 0.001), these variables were analyzed by two different Cox models to avoid collinearity. The presence of SABR and BED ≥65 Gy correlated significantly with superior OS in separate multivariable analysis models (Table 3). Furthermore, we identified that the SABR group with BED ≥65Gy showed a higher survival rate than the CFRT group with BED < 65Gy (*p* = 0.005) (Fig. 5).

**Table 3:** Univariable and multivariable analysis of the entire cohort

	Univariable		Multivariable (model 1) †		Multivariable (model 2) †	
Variable	HR (95% CI)	p	HR (95% CI)	p	HR (95% CI)	p
SABR vs. CFRT	0.615 (0.410–0.924)	0.019	0.623 (0.427–0.909)	0.014		
BED < 65 vs. ≥ 65	1.482 (1.039–2.115)	0.030			1.682 (1.150–2.462)	0.007
Time of treatment Before December 31, 2011 vs. after December 31, 2011	1.008 (0.706–1.439)	0.965				
Age ≤ 60 vs. > 60	0.882 (0.586–1.327)	0.547				
Sex male vs. female	1.094 (0.687–1.741)	0.706				
ECOG 0–1 vs. 2	0.554 (0.315–0.977)	0.041	0.523 (0.312–0.877)	0.014	0.572 (0.342–0.957)	0.033
Extrahepatic metastasis yes vs. no	1.731 (0.860–3.484)	0.124				
AFP ≤200 vs. > 200	0.606 (0.424–0.866)	0.006	0.566 (0.378–0.846)	0.006	0.520 (0.344–0.785)	0.002
Child-Pugh class A vs. B	0.383 (0.249–0.588)	< 0.001	0.560 (0.381–0.823)	0.003	0.511 (0.345–0.756)	0.001
Prior Treatment yes vs no	0.690 (0.483–0.986)	0.042	0.955 (0.647–1.410)	0.816	0.903 (0.607–1.343)	0.615
Single vs. multiple	0.379 (0.233–0.617)	< 0.001	0.494 (0.323–0.756)	0.001	0.474 (0.308–0.729)	0.001
Tumor size ≤8 vs. > 8	0.452 (0.296–0.689)	< 0.001	0.538 (0.360–0.804)	0.002	0.550 (0.367–0.825)	0.004
Vp3 vs. Vp4	0.641 (0.426–0.964)	0.033	0.678 (0.467–0.985)	0.041	0.685 (0.471–0.995)	0.047
Sorafenib yes vs. no	0.980 (0.685–1.401)	0.911				

*Abbreviations: SABR* Stereotactic ablative radiotherapy, *BED* Biologically effective dose, *ECOG* Eastern Cooperative Oncology Group, *EM* Extrahepatic metastasis, *AFP* Alpha feto protein, *HR* Hazard ratio, *CI* Confidence interval

†BED correlated highly with radiotherapy technique. Two Cox models were used to avoid collinearity

### Toxicity

Acute toxicities observed in the groups are shown in Table 4. Fatigue was the most common adverse event in both groups. Grade 3 abdominal pain (*n* = 1) in the SABR group, and grade 3 diarrhea (*n* = 2) in the CFRT group were recorded. One patient in CFRT group presented with a treatment-related grade 5 duodenal ulcer.

**Table 4:** Acute toxicity observed due to SABR and CFRT in the entire cohort

	SABR			CFRT			
	No. of patients (%)			No. of patients (%)			
	Grade 1	Grade 2	Grade 3	Grade 1	Grade 2	Grade 3	Grade 5
Nausea	5(9.3)	2(3.7)		9(10.5)	3(3.5)		
Vomiting	3(5.6)	1(1.8)		2(2.3)	1(1.2)		
Abdominal pain	9 (16.7)	2(3.7)	1(1.9)	6(7.0)			
Diarrhea	1(1.9)	1(1.9)		6(7.0)	4(4.7)	2(2.3)	
Fatigue	11 (20.3)	2(3.7)		17 (19.8)	1(1.2)		
Anorexia	10 (18.5)	2(3.7)		10 (11.6)	1(1.2)		
Duodenal ulcer		2(3.7)			1(1.2)		1(1.2)

*Abbreviations: SABR* Stereotactic ablative radiotherapy, *CFRT* Conventionally fractionated radiotherapy

Seven patients (13%) in the SABR group and 11 patients (12.8%) in the CFRT group experienced non-classic RILD, while two patients (3.7%) in the SABR group and six patients (7%) in the CFRT group experienced classic RILD. The incidences of RILD were not different between groups, even after pooling RILD types (16.7% vs. 19.8%, *p* = 0.646). There were no RILD–related deaths. After excluding 23 patients with missing follow-up CP scores, 10 patients (22.2%) in the SABR group and 19 patients (26.4%) in the CFRT group experienced an increase of CP score ≥ 2, which was not statistically different between groups (*p* = 0.612).

## Discussion

Although CFRT has benefits in PVI, the long-term outcome is still poor. Thus, more effective and promising treatments like SABR need to be explored. Unlike previous CFRT series, most SABR series have included few or no patients with PVI; the efficacy of SABR in PVI is thus not clear. A few SABR series including only PVI patients have reported a 1-year OS of 43.2–50.3% and an ORR of 44.4–86.3% [18–20]. Bujold et al. [21] have conducted the largest prospective trial of SABR for advanced HCC with 112 patients, of whom 55% presented with portal vein thrombosis (PVT). The overall 1-year OS was 44%, but the outcomes of the PVT subgroup were not reported.

Until now, direct head-to-head comparisons between different RT techniques in HCC with PVI have remained rare and controversial. In a cohort of patients with either PVT or inferior vena cava tumor thrombosis, Matsuo et al. [22] treated 43 patients with SABR (27 with CyberKnife and 16 with TrueBeam) and 54 patients with CFRT. The 1-year OS rate with SABR (using CyberKnife) was significantly higher than that with CFRT (56.7% vs. 29.3%, *p* = 0.02); similar trends were observed with local control and tumor response. In the meta-analysis performed by Rim et al. [23], SABR did not improve survival rates relative to CFRT in PVT patients. With only single-arm studies enrolled in their analysis, pooled estimates may not reveal the true head-to-head comparison as the heterogeneous designs and populations among studies. In the present study, the survival rates appear to be inferior to the published pooled results [23], in which the tumor size is much smaller than our cohort, with a median tumor size of 1.5–2.5 cm in the published pooled SABR cohort when compared with the median tumor size of 7.8 cm in our SABR cohort. However, when we analyzed only the outcome of tumors with ≤8 cm (median size: 5.1 cm) in our SABR series, the 1-year OS was 50% (data not shown), which is comparable to the 1-year OS of 48.5% in the pooled results. Tumor characteristics might partly explain the survival difference. Since some other factors affect survival as well, propensity score matching in the present study showed that SABR was superior to CFRT in terms of ORR, OS, and IFPS.

Some studies reported a higher prescribed dose or BED was correlated with better LC or survival [24–26]. According to our multivariable model, a BED ≥65 Gy was found to be a predictor of survival prolongation. We further used a ROC curve to define optimal cut-off points for separate groups. Interestingly, higher BED was associated with better survival and ORR in the CFRT group, while no optimal cut-off point was found in the SABR group. In a CFRT series, Kim et al. [27] have reported that PVT patients receiving BED ≥58 Gy had a higher response rate than did those receiving BED < 58 Gy (54.6% vs. 20%, *p* = 0.034). Toya et al. [9] have also reported that BED (< 58 vs. ≥58 Gy) was a significant predictor of tumor response and survival. In one pooled analysis of SABR, Ohri et al. [28] reported that there was no dose response relationship in terms of local control when treating primary liver tumors, which is comparable to our findings. The narrow dose range and small sample size in our study may have led this result. We reasoned that higher objective response resulting from adequate BED contributes to survival benefit. In agreement, a previous hypothesis states that RT may reduce or stabilize PVI, leading to the restoration of vascular flow and slowing down of intrahepatic tumor dissemination, thus halting the deterioration of hepatic function.

Historically, RT has not been widely used for liver tumors because of a significant risk of RILD, though modern RT techniques have reduced this risk. A RILD incidence of 8–19% for CFRT [29, 30] and 0–5% for SABR [31, 32] has been reported. Moderately higher RILD incidence in our cohort may be explained by less favorable populations included, hepatitis B virus prevalence, and challenge to discern the causes of liver enzyme elevation. In agreement with our findings, CP score increase (another endpoint) was reported in 10 to 30% of primary liver cancer patients after SABR treatment [21, 33]. We chose these endpoints for robust data comparison. In our study, SABR and CFRT had comparable RILD incidences and CP score increases, which was possibly attributable to a relatively small cohort or the similar constraints (with 700 ml normal liver < 15 Gy) applied in both techniques. Even so, SABR leads to an improvement in therapeutic index, as it gives a greater level of clinical benefit for the same level of morbidity.

The high possibility of failure outside the radiation field implies the necessities for combining regional or systemic treatments with RT. Numerous combinations have been reported, though most were retrospective and poorly-evidenced. A recently-published randomized-controlled trial conducted by Yoon et al. [34] demonstrated that compared to sorafenib, TACE+3DCRT yielded higher progression-free survival, time to progression, ORR, and OS in HCC with macroscopic vascular invasion. We were unable to detect differences between TACE+SABR versus TACE+CFRT in subgroup analysis in our study. Given the potential superiority of SABR, replacement of CFRT with SABR is a consideration for future combination investigations.

The primary limitation of this study is the retrospective, single-institutional design. Even though propensity score matching minimizes the bias related to treatment assignment, unmeasured confounding may have existed. Further prospective studies are warranted. We note that issues regarding state-of-the-art technology may have affected treatment outcomes. However, we grouped patients into 2 treatment periods in the statistical analysis, which may eliminate this concern.

## Conclusion

In summary, we demonstrated that compared to CFRT, SABR led to superior ORR, OS, and IFPS in propensity score-matched PVI patients. SABR delivers higher BED without increasing hepatic toxicities, and hence is a suitable RT modality for PVI patients. Further studies are required to validate our results.

## Data Availability

The datasets generated and analyzed during the current study are available from the corresponding author on reasonable request.

## Abbreviations

3DCRT: Three dimensional conformal radiotherapy
AFP: Alpha-fetoprotein
BED: Biologically effective dose
CFRT: Conventionally fractionated radiotherapy
CP: Child-Pugh
CT: Computed tomography
CTV: Clinical target volume
ECOG: Eastern Cooperative Oncology Group
HCC: Hepatocellular carcinoma
IFPS: In-field progression-free survival
IQR: Inter-quartile range
MRI: Magnetic resonance imaging
ORR: Overall response rate
OS: Overall survival
PTV: Planning target volume
PVI: Portal vein invasion
PVT: Portal vein thrombosis
RILD: Radiation-induced liver disease
ROC: Receiver operating characteristic
RT: Radiotherapy
SABR: Stereotactic ablative radiotherapy
TACE: Transarterial chemoembolization
